# Periinterventional inflammation and blood transfusions predict postprocedural delirium after percutaneous repair of mitral and tricuspid valves

**DOI:** 10.1007/s00392-021-01886-z

**Published:** 2021-06-01

**Authors:** Maria I. Körber, Matthieu Schäfer, Rakave Vimalathasan, Victor Mauri, Christos Iliadis, Clemens Metze, Henrik ten Freyhaus, Volker Rudolph, Stephan Baldus, Roman Pfister

**Affiliations:** 1 Department of Internal Medicine III, Division of Cardiology, Pneumology, Angiology and Intensive Care, University of Cologne, Kerpener Str. 62, 50937 Cologne, Germany; 2 Clinic for General and Interventional Cardiology/Angiology, Herz- und Diabeteszentrum NRW, Ruhr-Universität Bochum, Bad Oeynhausen, Germany

**Keywords:** Percutaneous repair of atrioventricular valves, Delirium, MitraClip

## Abstract

**Objectives:**

The aim of this study was to examine predictors and impact of postoperative delirium (POD) on outcome after percutaneous repair of mitral and tricuspid valves.

**Background:**

POD is common in elderly patients and contributes to increased health care costs and worse outcome. Predictors of POD in percutaneous mitral or tricuspid valve procedures are unclear.

**Methods:**

In a prospective single-center study, patients were screened for POD using the Confusion Assessment Method on the first and second postprocedural days, and up until 7 days in patients with clinical suspicion of delirium. Associations of POD with baseline characteristics, periprocedural outcome and mid-term mortality were examined.

**Results:**

One hundred and seventy-seven patients were included (median age 78 years [72–82], 41.8% female) and median (IQR) follow-up was 489 (293–704) days. Patients developing POD (*n* = 16, 9%) did not differ in baseline and procedural characteristics but more often received postinterventional blood transfusions (37.5% vs. 9.9%, *p* value = 0.007) and suffered from infections (43.8% vs. 9.9%, *p* value = 0.001). Patients with POD showed worse survival (HR: 2.71 [1.27–5.78]; *p* = 0.01), with an estimated 1-year survival of 46 ± 13% compared to 80 ± 3% in patients without POD (log-rank *p* value 0.007). In multivariate Cox regression, POD remained a significant predictor of mid-term mortality (HR 4.75 [1.97–11.5]; *p* = 0.001).

**Conclusion:**

After percutaneous mitral or tricuspid valve repair, POD was independently associated with worse mid-term survival. Procedure- rather than patient-associated characteristics such as blood transfusions and infections emerged as important risk factors for development of POD. Considering the substantial prognostic impact of POD, further studies on its prevention are warranted to improve patient outcome.

## Introduction

Delirium is a common organic brain syndrome with an acute onset of neurocognitive dysfunction. The pathophysiology of delirium is complex and not yet fully understood [1]. However, the interplay between preexisting morbidity and precipitating noxious insults such as major medical interventions can finally cause imbalance of brain chemistry and cerebral dysfunction resulting in postoperative delirium (POD). Elderly people and patients with functional impairment and complex multimorbidity seem to be especially vulnerable [2]. Hospitalized seniors are diagnosed with delirium in up to 50% and the number of unreported cases may be even higher, since delirium is easily overlooked in clinical routine [3, 4]. Delirium substantially contributes to health care costs through increased resource utilization and prolonged hospitalization. A study in the United States calculated that the 30-day cumulative cost attributable solely to delirium in patients on the intensive care unit is 17,838 US Dollar [5]. Furthermore, delirium is associated with worse survival as well as higher risk for functional and cognitive decline in the elderly [6, 7]. Since it is potentially preventable, delirium is also an important target for supportive interventions to improve patient outcome [8].

During recent years, the number of percutaneous procedures for treatment of valvular heart disease has steadily increased [9]. According to current guidelines, criteria for catheter-based treatment approaches are advanced age, frailty, functional disabilities and extensive organic morbidity [10]. These factors make patients prone to develop POD. Patients with mitral or tricuspid valve regurgitation undergoing percutaneous valve treatment often show functional etiology and advanced heart failure which is also associated with POD risk [11]. Recently, we reported an incidence of POD of 9% after such procedures and a strong association with prolonged postprocedural recovery and short-term survival [12]. With this study, we sought to investigate clinical predictors of POD and impact on mid-term mortality.

## Methods

### Study design and patient population

We conducted an observational, prospective cohort study. All consecutive patients undergoing percutaneous mitral or tricuspid valve repair at the Heart Centre of the University of Cologne between November 2017 and May 2019 were eligible for inclusion. Patients who denied consent or had significant language barrier were excluded. Preprocedural evaluation of cognitive function was performed using the Short Portable Mental Status Questionnaire. Patients with severe cognitive impairment (8 or more errors) were excluded. All patients were discussed in an interdisciplinary heart team and percutaneous therapy was decided based on individual surgical risk. Valve repair procedures using the Cardioband (Edwards Lifescience), Pascal (Edwards Lifescience) and MitraClip (Abbott) device have been described in detail elsewhere [13–15]. All procedures at our institution were conducted under general anesthesia, guided by transesophageal echocardiography and used transfemoral access. All patients provided written informed consent. The study was approved by the local ethics committee of the University of Cologne (14-116).

Baseline demographic and clinical characteristics were retrieved either from medical records or an automated information system (ORBIS, Agfa Healthcare, Bonn, Germany). For functional assessment, New York Heart Association (NYHA) functional class and Minnesota Living with Heart Failure Questionnaire (MLWHFQ) were evaluated on the day before the intervention. Frailty was evaluated according to the criteria defined by Fried et al. as previously reported [16]. Complications assessed according to the Mitral Valve Academic Research Consortium were all-cause mortality, neurological events, acute kidney injury, access-related vascular complications, major cardiac structural complications related to access, and technical success [17]. We defined procedural success as technical success and reduction of regurgitation to grade ≤ 2. Bleeding events were defined on a functional basis as a periprocedural drop (up until 72 h after procedure) of hemoglobin ≥ 3 g/dl or a periprocedural drop of hemoglobin with blood transfusion, regardless of bleeding site or direct clinical impact since anemia per se might contribute to the development of delirium. Infection was defined as clinically overt signs of infection with simultaneous increase in C-reactive protein or leucocyte count that needed attending of the treating physician, when recorded within 72 h after the procedure. Follow-up was assessed about 6 weeks after the initial procedure and included NYHA functional class and MLWHFQ. Mortality data were retrieved in May 2020, 1 year after the last patient was included. Patients or their general practitioner were contacted by phone.

### Assessment of POD

POD was assessed using a 2-step approach in line with current recommendations [18]: first, the Richmond Agitation Sedation Scale (RASS) was used as a valid and reliable tool to evaluate sedation and arousal on a 10-point scale [19]. In the case of RASS score of − 4 or − 5 (comatose state without reaction to verbal stimulation) POD was reassessed at a later time point. If patients had a RASS score of − 3 or higher, the Confusion Assessment Method for the Intensive Care Unit (CAM-ICU) was used as second step. CAM-ICU is assessing delirium based on 4 features derived from the Diagnostic and Statistical Manual of Mental Disorders [20]: acute onset or fluctuating course of mental status change (1), inattention (2), disorganized thinking (3) and altered level of consciousness (4). It is considered positive if both features (1) and (2) plus either feature (3) or (4) are present [21, 22]. POD was assessed on the first and second postoperative day for every patient by trained study staff, and additional assessment up to 7 days after the initial procedure in case of suspected delirium by the treating nurse or attending physician. Delirium was considered present if at least 1 CAM-ICU assessment was positive during the study period. At our center, there was no standard operating procedure for delirium prevention or treatment at the time of the study. In general, early mobilization was supported after the intervention and medical treatment was used according to the discretion of the physician in charge.

### Statistical analysis

Normally distributed continuous variables are expressed as mean ± standard deviation and the Student *t* test was used to compare patients with and without POD. If not normally distributed, continuous variables are expressed as median with interquartile range and Mann–Whitney *U* Test was used to calculate the statistical significance of differences by subgroup. Nominal and ordinal data are expressed as percentages and the statistical significance of differences was calculated using the Chi-square test. If the expected value in any of the cells was < 5, the Fisher exact test was used. Logistic regression analysis was used to estimate the odds ratio associated with predictors of POD. To identify risk factors of mortality, uni- and multivariate Cox regression models were fitted. Significant variables in univariate analysis were forwarded to the multivariate model. Only one variable concerning bleeding complications and one variable concerning functional status was included in the same multivariate model. Multivariate Cox regression used a stepwise backward elimination procedure retaining all variables significant at the *p* ≤ 0.10 level. Survival curves were estimated using Kaplan–Meier method and compared by log-rank test. Observations were censored at date of death or last confirmed status alive. All tests were 2-tailed and a *p* value < 0.05 was considered to indicate statistical significance. All statistical analyses were performed using SPSS Statistics for Windows, version 25 (IBM Corp., Armonk, NY).

## Results

One hundred and eighty-seven patients were eligible for study inclusion. Six patients were excluded due to missing informed consent. Four additional patients were excluded due to language barrier, leaving 177 patients for analysis. Of these 78% (*n* = 138) underwent percutaneous repair of the mitral valve using MitraClip and 2.8% (*n* = 5) using PASCAL. Five patients (2.8%) underwent percutaneous edge-to-edge repair of the tricuspid valve and ten patients (5.6%) underwent simultaneous edge-to-edge repair of mitral and tricuspid valve. Twelve patients (6.8%) underwent direct annuloplasty of the mitral and 7 (4%) patients of the tricuspid valve with the Cardioband. Baseline characteristics of patients are shown in Table 1. As previously shown, the overall incidence of POD was 9% (*n* = 16) and patients with versus without POD did not differ significantly with respect to comorbidities, functional parameters or technical/procedural success (Tables 1 and 2).

**Table 1:** Baseline characteristics of the study population stratified by occurrence of postoperative delirium

	Overall (*n* = 177)	Delirium (*n* = 16)	No delirium (*n* = 161)	*p* value
Age, years	78 (72–82)	81 (74–83)	78 (71–82)	0.34
Male	103 (58)	7 (44)	96 (60)	0.22
BMI, kg/m^2^	25 (22–29)	24 (23–28)	25 (22–29)	0.94
Cause of regurgitation				0.42
Functional	59 (47)	4 (31)	55 (49)	
Degenerative	38 (30)	5 (39)	33 (30)	
Combined pathology	28 (22)	4 (31)	24 (21)	
EuroScore II, %	6 (3–11)	4 (2–9)	6 (4–12)	0.11
NYHA functional class III–IV	152 (86)	13 (81)	139 (87)	0.51
LVEF (%)	43 (30–58)	54 (25–57)	43 (30–58)	0.65
Estimated GFR, ml/min	42 (32–56)	42 (30–58)	42 (32–56)	0.96
Hemoglobin, g/dl	12.1 ± 2.0	11.9 ± 1.6	12.1 ± 2.1	0.74
Anemia (♂ Hb < 13.6 g/l ♀ Hb < 12.0 g/l)	104 (59)	8 (50)	96 (60)	0.46
Diabetes mellitus	44 (25)	3 (19)	41 (26)	0.76
Hypertension	131 (74)	10 (63)	121 (75)	0.37
COPD	24 (14)	3 (19)	21 (13)	0.46
Prior stroke/TIA	28 (16)	3 (19)	25 (16)	0.72
Coronary artery disease	103 (58)	11 (69)	92 (57)	0.37
Atrial fibrillation	135 (76)	14 (88)	121 (75)	0.37
Previous cardiac surgery	58 (33)	5 (31)	53 (33)	0.89
Depression	10 (6)	0 (0)	10 (6)	0.60
Parkinson’s disease	2 (1)	0 (0)	2 (1)	1.00
Moderate cognitive impairment (5–7 errors in SPMSQ)	4 (2)	1 (6)	3 (2)	0.32
Smoking status				0.55
Never smoker	65 (37)	7 (47)	58 (36)	
Prior smoker	100 (57)	7 (47)	93 (58)	
Current smoker	11 (6)	1 (7)	10 (6)	
Frailty	73 (42)	7 (47)	66 (42)	0.71
MLWHFQ score	43 ± 21	43 ± 24	43 ± 21	1.00

Values are *n* (%), mean ± standard deviation or median (interquartile range)

*BMI* body mass index, *LVEF* left ventricular ejection fraction, *GFR* glomerular filtration rate, *Hb* hemoglobin, *COPD* chronic obstructive pulmonary disease, *TIA* transient ischemic attack, *SPMSQ* Short Portable Mental Status Questionnaire, *MLWHFQ* Minnesota Living With Heart Failure Questionnaire

**Table 2:** Procedural characteristics by occurrence of postoperative delirium

	Overall (*n* = 177)	Delirium (*n* = 16)	No delirium (*n* = 161)	*p* value
Intervention				
MV clip	138 (78)	14 (88)	124 (77)	0.53
TV clip	5 (3)	0	5 (3)	1.00
MV + TV clip	10 (6)	0	10 (6)	0.60
Cardioband MV	12 (7)	0	12 (8)	0.61
Cardioband TV	7 (4)	0	7 (4)	1.00
PASCAL	5 (3)	2 (13)	3 (2)	0.07
Technical success	172 (97)	16 (100)	156 (97)	1.00
Procedural success	146 (83)	14 (88)	132 (82)	0.74
Procedural duration, min	143 (113–201)	135 (103–173)	144 (114–206)	0.39
Use of intravenous anesthesia	43 (25)	6 (43)	37 (24)	0.20
Periprocedural stroke/TIA	0 (0)	0 (0)	0 (0)	1.00
Major access-related vascular complications	0 (0)	0 (0)	0 (0)	1.00
Acute kidney injury	5 (3)	0 (0)	5 (3)	1.00
Mechanical ventilation > 48 h	6 (3)	2 (13)	4 (3)	0.09

Values are n (%) or median (interquartile range)

*MV* mitral valve, *TV* tricuspid valve, *TIA* transient ischemic attack

**Fig. 1:**
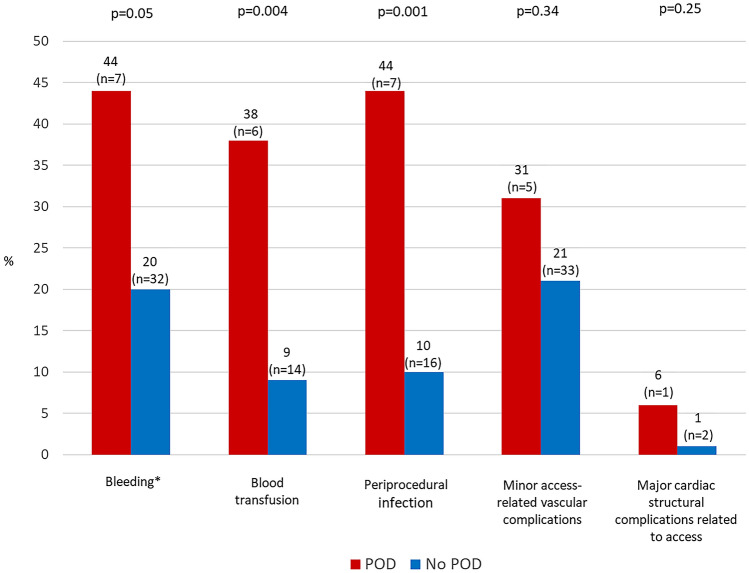
Periprocedural complications by occurrence of postoperative delirium. Bars show the rate of periprocedural complications by occurrence of postoperative delirium as % (*n*). *p* value for comparison of patients with and without POD. *POD* postoperative delirium. *Bleeding was defined as a periprocedural drop (up until 72 h after procedure) of hemoglobin ≥ 3 g/dl or a periprocedural drop of hemoglobin with blood transfusion, regardless of bleeding site

Figure 1 shows periprocedural complications by development of POD. Postprocedural infection was more frequent in patients developing POD (43.8% vs. 9.9%, *p* value = 0.001), with a 7.05-fold (95% CI 2.31–21.5) increased odds of POD. Patients who developed POD did receive blood transfusions more often (37.5% vs. 8.7%, *p* value = 0.004), with a 6.30-fold (1.99–19.9) increased odds of POD. Two of the patients with transfusion had bleeding associated with the access site, two with the central venous catheter and two with other localisations (gastrointestinal and pharyngeal). The remaining blood transfusions (*n* = 14) were not accompanied by clinically overt bleeding but due to a relevant drop in hemoglobin level. A prolonged mechanical ventilation was more common in patients with POD, but this was not statistically significant (Table 2).

Hospital stay and stay on intensive care unit were longer in patients suffering from POD. Functional improvement at 6 weeks was not different between groups (Table 3).

**Table 3:** Clinical outcome parameters

	Overall (*n* = 177)	Delirium (*n* = 16)	No delirium (*n* = 161)	*p* value
Periprocedural death	7 (4)	2 (13)	5 (3)	0.13
Length of hospital stay, days	6 (5–8)	8 (6–19)	6 (4–8)	0.01
Length of stay on ICU, days	1 (1–2)	4 (2–9)	1 (1–2)	< 0.001
Improvement in MLWHFQ score	6 (− 1 to 17) (*n* = 137)	10 (− 1 to 22)	5 (− 1 to 17)	0.43
Improvement ≥ 1 NYHA classes	40 (30)	3 (30)	37 (30)	1.00

Values are *n* (%) or median (interquartile range). ICU = intensive care unit, MLWHFQ = Minnesota Living With Heart Failure Questionnaire

**Fig. 2:**
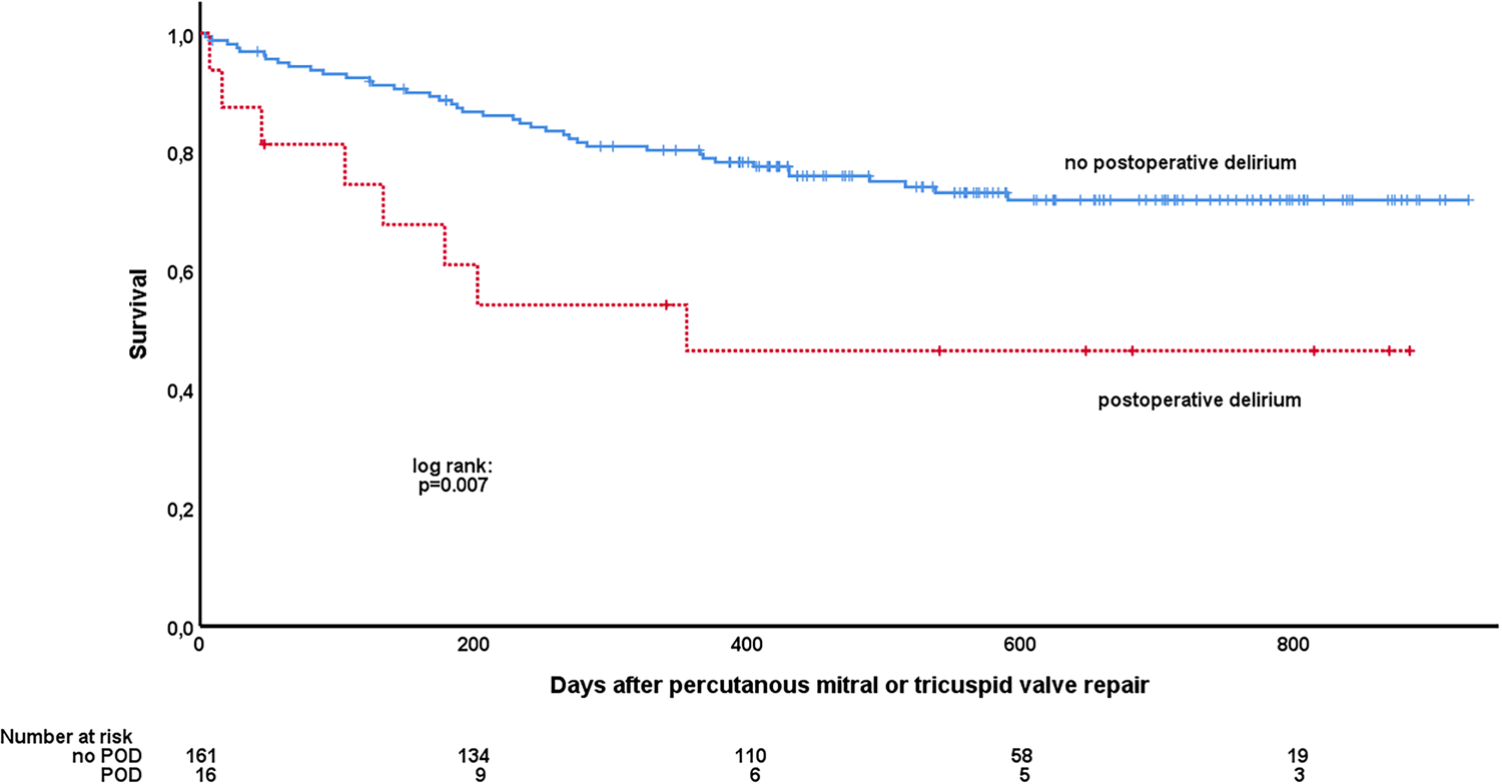
Kaplan–Meier survival estimates stratified by postoperative delirium. Kaplan–Meier curves comparing patients with versus without delirium are shown. *p* value is derived from log-rank test. *POD* postoperative delirium

Median follow-up time was 489 (293–704) days. Mortality rate of the total population during follow-up was 28% (*n* = 49). The occurrence of POD was significantly associated with worse survival (HR of mortality 2.71 [1.27–5.78]; *p* = 0.01]). Estimated survival at 1 year was 80 ± 3% in patients without POD and 46 ± 13% in patients with POD (Fig. 2, log-rank *p* value 0.007). When adjusting for significant covariates POD remained significantly associated with mortality (HR 4.75, 95% CI 1.97–11.46, *p* = 0.001) (Table 4). Replacing MLWHFQ score with frailty or NYHA functional class or hemoglobin with blood transfusion did not significantly change results.

**Table 4:** Cox regression models for all-cause mortality

	Univariate cox-regression		Multivariate cox-regression	
	HR (95% CI)	*p* value	HR (95% CI)	*p* value
POD	2.71 (1.27–5.78)	0.010	4.75 (1.97–11.46)	0.001
Age, years	1.00 (0.97–1.03)	0.832		
Male	1.87 (1.02–3.43)	0.044		N.S
BMI, kg/m^2^	0.95 (0.89–1.01)	0.090		
Cause of regurgitation				
Functional				
Degenerative	0.77 (0.31–1.91)	0.575		
Combined pathology	1.28 (0.54–3.06)	0.574		
EuroScore II, %	1.01 (0.99–1.04)	0.338		
NYHA functional class III–IV	4.51 (1.094–18.556)	0.037		^b^
LVEF (%)	0.99 (0.97–1.00)	0.115		
Estimated GFR, ml/min	0.99 (0.98–1.01)	0.220		
Hemoglobin, g/dl	0.70 (0.61–0.81)	< 0.001	0.08 (0.68–0.94)	0.005
Anemia (♂ Hb < 13.6 g/l ♀ Hb < 12.0 g/l)	2.93 (1.50–5.73)	0.002		
Diabetes mellitus	1.96 (1.01–3.50)	0.024		N.S
Hypertension	0.90 (0.49–1.68)	0.749		
COPD	0.84 (0.36–1.97)	0.681		
Prior stroke/TIA	1.05 (0.49–2.23)	0.907		
Coronary artery disease	1.31 (0.73–2.34)	0.368		
Atrial fibrillation	0.967 (0.50–1.85)	0.919		
Previous cardiac surgery	1.38 (0.78–2.45)	0.275		
Depression	0.808 (0.20–3.33)	0.768		
Parkinson’s disease	0.05 (0.00–7177.22)	0.619		
Moderate cognitive impairment (5–7 errors in SPMSQ)	0.76 (0.11–5.51)	0.686		
Smoking status				
Never smoker				
Prior smoker	3.14 (1.46–6.76)	0.003	4.28 (1.74–10.56)	0.002
Current smoker	3.43 (1.03–11.39)	0.044	4.71 (1.14–19.50	0.032
Frailty	2.31 (1.28–4.15)	0.005		^b^
MLWHFQ score	1.02 (1.00–1.03)	0.011	1.02 (1.00–1.04)	0.017
Technical success	1.30 (0.18–9.43)	0.80		
Procedural success	0.39 (0.21–0.72)	0.003	0.35 (0.18–0.67)	0.002
Procedural duration, min	1.00 (1.00–1.01)	0.25		
Use of intravenous anesthesia	0.91 (0.46–1.79)	0.79		
Periprocedural stroke/TIA	Group too small			
Minor access-related vascular complications	0.97 (0.48–1.94)	0.93		
Major access-related vascular complications	Group too small			
Major cardiac structural complications related to access	Group too small			
Bleeding^a^	1.41 (0.75–2.65)	0.29		
Blood transfusion	3.03 (1.51–6.01)	0.002		^b^
Periprocedural infection	1.42 (0.69–3.14)	0.32		
Acute kidney injury	0.73 (0.101–5.31)	0.76		
Mechanical ventilation > 48 h	1.40 (0.34–5.79)	0.64		

*HR* hazard ratio, *POD* postoperative delirium, *BMI* body mass index, *LVEF* left ventricular ejection fraction, *GFR* glomerular filtration rate, *Hb* hemoglobin, *COPD* chronic obstructive pulmonary disease, *TIA* transient ischemic attack, *SPMSQ* Short Portable Mental Status Questionnaire, *MLWHFQ* Minnesota Living With Heart Failure Questionnaire, *N.S.* not significant

^a^Bleeding was defined as a periprocedural drop (up until 72 h after procedure) of hemoglobin ≥ 3 g/dl or a periprocedural drop of hemoglobin with blood transfusion, regardless of bleeding site

^b^Only one variable concerning bleeding complications and one variable concerning functional status was included in the same multivariate model. Replacing MLWHFQ score with frailty or NYHA functional class or hemoglobin with blood transfusion did not significantly change results

## Discussion

Here, we extend our earlier observations on incidence and short-term prognostic impact of POD in patients undergoing percutaneous repair of mitral or tricuspid valves. We identified postprocedural infection and blood transfusion as clinical predictors of POD. Patients suffering from POD had an at least threefold increased mortality during mid-term follow-up and the association between POD and mortality remained significant after adjusting for relevant risk factors.

### Predictors of POD

The identification of predictors of POD, particularly if modifiable, is of major clinical interest as a target for preventive interventions. However, POD shows a complex multifactorial pathophysiology [2] and predictors of POD might strongly differ by type of procedure and patient characteristics. For example, carotid artery disease and atrial fibrillation are predictors of POD after cardiac surgery and TAVR [23] suggesting that POD may be triggered by subclinical ischemic brain injury resulting from vascular or cardiac microemboli [24]. Transcatheter tricuspid valve procedures are not within the systemic circulation which precludes the latter pathophysiological pathway. Transcatheter mitral valve procedures do not include arterial vascular manipulations and latent thrombi in the left atrial appendage as potential origins of embolic events are generally excluded by transoesophageal echocardiography at the beginning of the procedure. This might explain why vascular disease and atrial fibrillation were not associated with POD in our cohort.

Our findings on the association of infections and blood transfusion with POD are plausible with respect to pathophysiology. The brain is particularly vulnerable to hypoxia, and a postprocedural drop in hemoglobin might contribute to cerebral perfusion deficit. In patients undergoing cardiac surgery the cerebral oxygen saturation is a known predictor of POD [25] and postoperative anemia as well as high blood transfusion count intraoperatively are known to be associated with POD in surgical patients [26, 27]. Postprocedural infection might also play a critical role for cerebral hypoxia and direct damage. A systemic inflammatory response causes a cascade of neuroinflammatory processes and impaired blood flow as well as neuronal apoptosis [2, 28].

Taken together, both risk factors of POD identified in our cohort might causally contribute to the development of POD and hence are of clinical value considering prevention. General protective measures to avoid common hospital-acquired infections such as early mobilization for prevention of pneumonia or early removal or omitting of urinary catheters for protection against urinary tract infections might be useful to prevent subsequent POD [29, 30]. Furthermore, routine postprocedural testing of inflammatory markers might be discussed for early detection and treatment initiation of clinical infections. Similarly, routine monitoring of hemoglobin levels might be helpful to detect a drop and prompt blood transfusions. However, the benefit of liberal blood transfusion for prevention of POD has to be demonstrated in future trials since existing evidence does not support this with regard to mortality outcomes [31].

### Clinical impact of POD

Our study shows that POD is of major clinical relevance in patients undergoing percutaneous mitral or tricuspid valve interventions. POD was associated with a more than threefold higher risk of death at 1 year. Our mid-term results extend our initial findings that already showed a negative prognostic trend of POD for survival at 30 days and 6 months [12]. This underlines the outstanding clinical relevance of POD [27, 32]. The question raises on whether POD is causal for mortality or serves as a surrogate parameter for otherwise vulnerable patients prone to worse outcomes. In support of the former, several pathophysiological mechanisms contributing to delirium (such as systemic hypoxia, metabolic abnormalities or stress responses within the sympathetic nervous system) also interfere with cellular metabolism and systemic inflammation [33] making a causal contribution plausible. However, the latter is usually regarded more likely and there are data showing that mortality in critically ill patients is not attributable to delirium alone [34]. Even if POD is not directly and causally linked with mortality, this is still of major relevance given that clinical surveillance and supportive interventions addressing impairments associated with POD might have the potential to improve patient outcome. For example, in non-demented community-dwelling elderly people rapid cognitive decline was associated with a doubled mortality risk [35]. Many patients undergoing transcatheter therapy of mitral or tricuspid valves suffer from heart failure. In heart failure patients cognitive impairment increases mortality risk by more than twofold which might be caused by weak treatment adherence [36]. Thus, despite the fact that the causal contribution of POD to mortality is not yet fully understood, the avoidance of POD seems highly important. Several non-pharmacological strategies exist to prevent POD including early mobilization, sleep–wake cycle preservation or cognitive stimulation activities [8, 37]. Further studies are warranted on possible prevention strategies and their effect in elderly patients undergoing mitral or tricuspid valve therapy. Prevention of POD might shorten length of hospital stay and eventually the risk for mid-term cognitive and functional decline with associated morbidity and mortality.

### Study limitations

We screened our patients using the CAM-ICU score, that is one of the most commonly used tools in routine assessment and recommended in current guidelines [18]. Advantages are the easy use, high specificity and inter-rater reliability [38]. Nevertheless, POD—especially when hypoactive—can easily be underestimated [4]. POD was systematically screened for only on the first 2 days after the procedure. On days 3–7, CAM-ICU was assessed only if clinical suspicion was raised by treating nurses or physicians, which might lead to a threefold underestimation of POD incidence [4]. However, the majority of POD occur early after interventions [32, 39] suggesting reasonable sensitivity of our approach. We only assessed selected outcomes such as in-hospital course and mortality up to 1 year. The clinical impact of POD might be even more pronounced in patient-centered outcomes such as independency in daily living and cognitive function. Nonetheless, there is already robust evidence from many other clinical settings on the association of POD with functional decline and independency in activities of living [27, 40].

## Conclusions

Postprocedural blood transfusion and infection emerged as relevant risk factors of POD following percutaneous repair of mitral or tricuspid valves. POD was associated with worse mortality at 1 year. These findings highlight the clinical impact of POD, and further study is warranted to evaluate preventive interventions for POD with the aim of improving patient outcome.

## Data Availability

Not applicable.
